# Health economics evaluation of screening for sarcopenia among community-dwelling older persons

**DOI:** 10.1186/s12889-025-25263-x

**Published:** 2025-11-07

**Authors:** Yang Liu, Boran Sun, Jingyue Wang, Zhanliang Ruan, Yuan Wang, Jian Sun, Shu Wang, Wenli Lu

**Affiliations:** 1 Department of Epidemiology and Statistics, School of Public Health, Tianjin Medical University, 22 Qixiangtai Road, Tianjin, 300070 China; 2 Department of General Medicine, Junliangcheng Hospital, Tianjin, 300301 China; 3 Tianjin Academy of Traditional Chinese Medicine Affiliated Hospital, Tianjin, 300120 China; 4 National Clinical Research Center for Chinese Medicine Acupuncture and Moxibustion, Tianjin, 300381 China

**Keywords:** Sarcopenia, Screening, Cost-effectiveness analysis, Quality-adjusted life-years

## Abstract

**Objective:**

Sarcopenia increased the risk of various adverse outcomes, including cardiovascular diseases, falls, and mortality. Implementing interventions could mitigate the harm caused by sarcopenia. With the dearth of health economics evaluation on sarcopenia screening, we aimed to evaluate the costs and benefits of sarcopenia screening strategies among community-dwelling older persons.

**Methods:**

We constructed a decision-analytic Markov model for a cohort of individuals aged 60 years and above with a total of 25 1-year cycles. A total of 20 screening strategies, with annual or biennial screening interval and 10 screening tools: SARC-F, MSRA-5, MSRA-7, calf circumference, Finger-ring test, Ishii test, SARC-CALF, AWGS 2019, SARC-F|MSRA-5 (Parallel test), and SARC-F|MSRA-7 (Parallel test), were assessed in our study. Cost-effectiveness analysis was conducted by calculating the cost, quality-adjusted life-years (QALYs) for each strategy. The corresponding incremental cost-effectiveness ratio (ICER) was obtained by comparing with no screening. Highly cost-effective strategies were considered if ICER was less than the per-capita GDP for China ($12,551.49), and the strategy with the lowest ICER was the most cost-effective. One-way deterministic and probabilistic sensitivity analyses (DSA & PSA) were used to assess the robustness of the main outcomes.

**Results:**

Compared with no screening, all screening strategies were highly cost-effective. The most cost-effective screening strategy was biennial SARC-F|MSRA-7 screening ($1461.52/QALY), followed by annual SARC-F|MSRA-7 screening ($2147.82/QALY), biennial AWGS 2019 screening ($2340.21/QALY), and annual AWGS 2019 screening ($2419.16/QALY). DSA results indicated that the model was relatively stable. PSA results indicated that the probability of annual AWGS 2019 screening being cost-effective increased to 100%, when the willing-to-pay threshold increased to $9800.

**Conclusion:**

Combining the PSA results, we recommended conducting annual sarcopenia screening with AWGS 2019 among community-dwelling older adults in China.

**Supplementary Information:**

The online version contains supplementary material available at 10.1186/s12889-025-25263-x.

## Introduction

Sarcopenia is an age-related geriatric syndrome characterized by a decline in muscle mass, reduction in muscle strength, and/or deterioration of physical function [1]. The decline in muscle mass would originate as early as early adulthood, starting with atrophy and loss of type II muscle fibers, and it would continue throughout life. The decline in muscle function would begin around the age of 35 and decrease at a rate of 1% to 2% per year. After the age of 60, the decline would accelerate, and after 75, the rate of decline would reach its peak [2–6].

Sarcopenia has caused significant health losses in the older persons. The global prevalence of sarcopenia among people aged 60 and above was approximately 10% [7]. It has been estimated that by 2050, the global number of individuals with sarcopenia would reach 200 million [1]. The prevalence of sarcopenia among older persons in Asia ranged from 5.5% to 25.7% [8]. In Chinese community-dwelling older persons, the prevalence of sarcopenia reached 12% [9]. Sarcopenia had insidious onset and progressed slowly [2, 3], but it could lead to adverse events such as decreased physical function, cognitive impairment, disability, and reduced quality of life [1, 10]. It was also associated with increased risks of cardiovascular disease (CVD) (*HR* 1.33, 95% *CI* 1.04–1.71) [11], falls (*OR* 1.89, 95% *CI* 1.33–2.68), fractures (*OR* 1.71, 95% *CI* 1.44–2.03) [12], hospitalization (*OR* 1.95, *P* < 0.001) [13], and mortality (*HR* 2.32, 95% *CI* 1.01–5.43) [14–16]. Falls, in particular, was the leading cause of injury-related deaths among individuals aged 65 and above in China [17]. Sarcopenia has imposed a heavy economic burden on the healthcare system and society. In the United States, the direct healthcare costs attributed to sarcopenia reached $18.5 billion in 2000, accounting for approximately 1.5% of total healthcare expenditures [18]. The total hospitalization costs for sarcopenic patients aged 65 and above amounted to $19.12 billion in 2014, and compared to individuals without sarcopenia, sarcopenic patients incurred an additional annual marginal cost of $2,315.7 per person [13].

The Asian Working Group for Sarcopenia (AWGS) 2019 consensus recommended the use of several screening tools for community-dwelling older persons, including the SARC-F questionnaire, SARC-F combined calf circumference (SARC-CALF) questionnaire, and calf circumference [8]. Studies have shown the detection rates for sarcopenia screening among community-dwelling older persons in South Korea using SARC-F (7.5%), SARC-CALF (26%), and calf circumference (43.5%) [19]. In Indonesia, the detection rates for sarcopenia screening among community-dwelling older persons were 6.5% (SARC-F), 16.1% (SARC-CALF), and 51.2% (calf circumference) [20]. In China, the detection rates for sarcopenia screening among community-dwelling older persons were 9.2% (SARC-F), 17.9% (SARC-CALF), 37.6% (calf circumference) [21], 28.5% (Mini Sarcopenia Risk Assessment, MSRA-7), and 29.4% (MSRA-5) [22]. In Japan, the detection rates for sarcopenia screening among community-dwelling older persons were 14.2% (males) and 22.1% (females) using the Ishii test [23], 15.4% (males) and 18.5% (females) using the Finger-ring test [24].

Few research conducted health economics evaluation of screening strategies for sarcopenia in the older persons. Only one study from Iran compared four screening strategies, including the screening by the European Working Group on Sarcopenia in Older People (EWGSOP 2010), SARC-F, MSRA-7, and the sarcopenia scoring assessment models (SarSA-Mod), with no screening. The results showed that all screening strategies were cost-effective, with the screening by the EWGSOP 2010 being the most cost-effective, with the incremental cost-effectiveness ratio (ICER) of $1875.67 per quality-adjusted life-years (QALYs) [25]. 

Therefore, this study aimed to evaluate and compare the cost-effectiveness of sarcopenia screening strategies among community-dwelling older persons, in order to provide decision-making support for the implementation and promotion of sarcopenia screening in this population in China.

## Methods

### Screening strategies

A total of 20 screening strategies, with annual or biennial screening interval and 10 screening tools, including SARC-F, MSRA-5, MSRA-7, calf circumference, Finger-ring test, Ishii test, SARC-CALF, AWGS 2019, SARC-F|MSRA-5, and SARC-F|MSRA-7, were assessed in our study.

### Model structure and assumptions

**Fig. 1:**
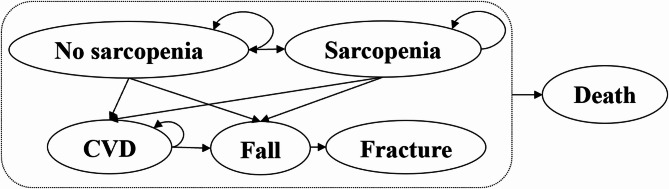
Markov model transition diagram of the natural history of sarcopenia

Based on previous studies [26], we constructed a decision-analytic Markov model for a cohort of individuals aged 60 years with a total of 25 1-year cycles [25], which provided a more comprehensive assessment of the occurrence and development of sarcopenia in their life course. The decision tree model was used to model the screening tool’s accuracy for sarcopenia. Based on the sensitivity and specificity of abovementioned screening tools, individuals were further classified into true positives, false negatives, true negatives, or false positives, respectively. As for a balance between model accuracy and computational complexity, we simulated the progression of 1,000 individuals [25] with considering CVD, falls, and fractures as complications or intermediate outcomes of sarcopenia, and death as the final outcome [27]. Therefore, the model included five states: no sarcopenia, sarcopenia, developed CVD, sarcopenia with CVD, and death (Fig. 1). The occurrence of CVD and fractures incurred corresponding treatment costs and reduced health utility values. Based on transition probabilities, 1,000 individuals could remain in the same state, progress to a disease state, or revert to a previous state in each year. All individuals were at risk of developing CVD, falls, or fractures.

Three assumptions were made in our study. Firstly, screening participation rate was assumed 100%. Secondly, all individuals with positive screening results would receive immediate intervention with a stable intervention effect. Thirdly, fractures could be cured within one year.

### Model input parameter

#### Transition probabilities

The parameters for the transition probabilities of each state, such as the incidence, prevalence, and mortality rates of CVD and fall, were obtained from the global burden of disease (GBD) 2019 (Supplemental Table 1). The fatality rate of CVD and fall-caused mortality rate used in this model were calculated as the ratio of the mortality rate to the prevalence rate for each disease within the corresponding age group. The prevalence of sarcopenia was derived from a meta-analysis among the Chinese community-dwelling older persons [9]. The mortality rate, incidence rate and recovery rate of sarcopenia as well as intervention effect parameters were all obtained from published literatures (Table 1).

**Table 1:** Main input parameters of CEA model

Variable	Value	SD/(CI)	Distribution	Sour
Transition probability				
Sarcopenia prevalence	0.12	0.10–0.15	beta	^[9]^
CVD increase the risk of falls	2.17	1.06–2.95	beta	^[28]^
Sarcopenia incidence rate				^[29]^
60–69	0.04			
70–79	0.12			
≥ 80	0.17			
Probability of death in non-sarcopenic individuals	0.09			^[25]^
Proportion of fractures after falls	0.26	0.23–0.29	beta	^[30]^
Sarcopenia increases the risk of developing CVD	1.33	1.04–1.71	beta	^[11]^
Sarcopenia increases the risk of fall	1.89	1.33–2.68	beta	^[12]^
Sarcopenia increases the risk of death	2.00	1.71–2.34	beta	^[31]^
Sarcopenia increases the risk of fracture	1.71	1.44–2.03	beta	^[12]^
Sarcopenia recovery rate	0.16	0.14–0.18	beta	^[26]^
Interventions reduce the risk of sarcopenia	0.45	0.37–0.55	beta	^[32]^
Interventions increase the probability of sarcopenia recovery	3.61	1.05–13.66	beta	^[33]^
The proportion of possible sarcopenia in non-sarcopenic individuals	0.40	0.36–0.44	beta	^[26]^
Sensitivity				
MSRA-7	0.81	0.72–0.88	beta	^[34]^
SARC-F	0.34	0.19–0.53	beta	^[35]^
Calf circumference	0.81	0.77–0.84	beta	^[35]^
MSRA-5	0.61	0.54–0.69	beta	^[35]^
Ishii test	0.81	0.78–0.85	beta	^[35]^
Finger-ring test	0.71	0.65–0.77	beta	^[35]^
SARC-F|MSRA-5	0.42	0.38–0.47	beta	^[36]^
SARC-F|MSRA-7	0.64	0.57–0.70	beta	^[36]^
SARC-CALF	0.59	0.57–0.70	beta	^[35]^
AWGS 2019	1.00			^[8]^
Specificity				
MSRA-7	0.43	0.35–0.52	beta	^[34]^
SARC-F	0.90	0.83–0.95	beta	^[35]^
Calf circumference	0.73	0.71–0.76	beta	^[35]^
MSRA-5	0.74	0.69–0.79	beta	^[35]^
Ishii test	0.76	0.73–0.79	beta	^[35]^
Finger-ring test	0.85	0.82–0.87	beta	^[35]^
SARC-F|MSRA-5	0.98	0.88–1.00	beta	^[36]^
SARC-F|MSRA-7	1.00			^[36]^
SARC-CALF	0.85	0.75–0.92	beta	^[35]^
AWGS 2019	1.00			^[8]^
Cost($)				
Annual discount rate	0.03	0.01–0.05	beta	^[37]^
Intervention	1119.05	± 119.05	gamma	^[25]^
Fracture treatment	3599.05	± 695.07	gamma	^[25]^
CVD treatment1^a^	4149.29	± 414.93	gamma	^[25]^
CVD treatment2^b^	1904.77	± 190.48	gamma	^[25]^
MSRA-7	0.71	± 0.07	gamma	^[25]^
SARC-F	0.71	± 0.07	gamma	^[25]^
MSRA-5	0.71	± 0.07	gamma	^[25]^
Calf circumference	0.21	± 0.02	gamma	S
Ishii test	1.52	± 0.15	gamma	^[25]^ S
Finger-ring test	0.21	± 0.02	gamma	S
SARC-F|MSRA-5	1.43	± 0.14	gamma	^[25]^
SARC-F|MSRA-7	1.43	± 0.14	gamma	^[25]^
SARC-CALF	0.92	± 0.09	gamma	S
Handgrip strength test	1.31	± 0.13	gamma	^[25]^
Gait speed test	0.71	± 0.07	gamma	^[25]^
DXA	42.86	± 4.29	gamma	^[25]^
Utilities				
Non-sarcopenic individuals	0.76	± 0.08	beta	^[25]^
Sarcopenic individuals	0.68	± 0.07	beta	^[25]^
Sarcopenic individuals with fracture	0.51	± 0.05	beta	^[25]^
Non-sarcopenic individuals with fracture				^[38, 39]^
60–64	0.57			
65–69	0.55			
70–74	0.53			
75–79	0.52			
80–84	0.51			
≥ 85	0.50			
Non-sarcopenic individuals with CVD	0.75	± 0.17	beta	^[40]^
Sarcopenic individuals with CVD	0.56	± 0.06	beta	^[25]^
Sarcopenic individuals with CVD and fracture	0.42	± 0.04	beta	^[25]^

*CVD* cardiovascular disease, *S* Survey, *DXA* Dual-energy X-ray absorptiometry

^a^The initial average per capita cost of treatment and care of cardiovascular patients

^b^ The incremental average per capita cost of treatment and care of cardiovascular patients

#### Utility and quality-adjusted life-years

The utility value represented weight for life quality in the particular health stage based on published literatures (Table 1). A utility value of 1 represented perfect health, while a value of 0 represented death. The occurrence of health impairments such as fractures or illnesses would lower the utility value. QALYs was thus obtained by multiplying the utility value by the life years lived in each health state which was a measurement to assess health effectiveness.

#### Cost

The costs in our study included screening costs and intervention costs for sarcopenia, fracture treatment costs, and CVD treatment costs. The cost parameters were mainly obtained from the research conducted by Darvishi A [25]. The labor costs for calf circumference and the Finger-ring test were calculated by using the labor cost calculation formula, based on the average annual salary of employees in China in 2021 [41]. All costs were discounted to the year 2021 with a discount rate of 3% [37].

#### Cost-effectiveness analysis

The ICER was used to evaluate the relative merits of each screening strategy compared with no screening. It was calculated by dividing the difference in costs between the screening strategy and no screening by the difference in their respective effects. Strategies were cost-effective if ICER were less than 3 times the per-capita gross domestic product (GDP) for China ($37,654.49). And strategies were considered highly cost-effective if ICER were less than the per-capita GDP for China ($12,551.49) [42]. The strategy with the lowest ICER was the most cost-effective.

#### Sensitivity analysis

Both deterministic sensitivity analysis (DSA) and probabilistic sensitivity analysis (PSA) were performed to examine the uncertainty of certain parameters in the model. Firstly, a tornado diagram was used to show the proportion of the influence of varying individual parameters on the model results (net benefit). The longer the bar chart in the tornado diagram, the higher the proportion of the influence [43]. Secondly, the model results were calculated using 1000 Monte Carlo simulations to conduct PSA, considering the distributions of each parameter (Table 1) and capturing the simultaneous variation of multiple variables. The PSA generated the cost-effectiveness acceptability curves (CEAC) and their corresponding reports. All statistical analyses were performed using TreeAge Pro.

## Results

### Base case analysis

The results showed that the Markov cohort’s healthy life expectancy was 69.0 years, which was close to the reported life expectancy of 68.5 years by the Chinese Center for Disease Control and Prevention in 2019 [44].

All screening strategies were highly cost-effective compared with no screening. The most cost-effective screening strategy was the biennial SARC-F|MSRA-7 screening ($1,461.52/QALY), followed by the annual SARC-F|MSRA-7 screening ($2,147.82/QALY), the biennial AWGS 2019 screening ($2,340.21/QALY), and the annual AWGS 2019 screening ($2,419.16/QALY). The biennial SARC-F|MSRA-5 screening strategy had the lowest average cost per person. The screening strategy with the highest gained QALY was the annual MSRA-7 screening (Table 2).

**Table 2:** Cost-effectiveness analysis of different sarcopenia screening strategies

Method	Interval	Cost ($)	Incr Cost ($)	Eff (QALY)	Incr Eff (QALY)	CE ($/QALY)	ICER ($/QALY)
No screening	-	10411.01	0.00	9.0011	0.0000	1156.64	-
SARC-F|MSRA-5	biennial	11001.03	590.02	9.2290	0.2279	1192.01	2588.90
SARC-F|MSRA-7	biennial	11064.42	653.41	9.4482	0.4471	1171.06	1461.52
SARC-F	biennial	11276.12	865.11	9.2061	0.2050	1224.86	4220.83
SARC-F|MSRA-5	annual	11398.31	987.30	9.3687	0.3677	1216.63	2685.39
SARC-F|MSRA-7	annual	11429.70	1018.70	9.4754	0.4743	1206.25	2147.82
AWGS 2019	biennial	11459.37	1048.36	9.4491	0.4480	1212.75	2340.21
SARC-CALF	biennial	11762.51	1351.50	9.3274	0.3263	1261.07	4141.59
Finger-ring test	biennial	11865.67	1454.66	9.3750	0.3739	1265.67	3890.64
AWGS 2019	annual	11904.95	1493.95	9.6186	0.6175	1237.70	2419.16
SARC-F	annual	11979.69	1568.69	9.3423	0.3412	1282.31	4597.49
MSRA-5	biennial	12312.81	1901.80	9.3584	0.3573	1315.70	5323.03
Ishii test	biennial	12395.75	1984.74	9.4302	0.4291	1314.47	4625.16
Calf circumference	biennial	12536.44	2125.43	9.4362	0.4351	1328.54	4884.42
SARC-CALF	annual	12783.55	2372.54	9.5015	0.5004	1345.42	4740.95
Finger-ring test	annual	12905.86	2494.85	9.5553	0.5542	1350.65	4501.77
MSRA-5	annual	13856.56	3445.56	9.5473	0.5462	1451.37	6308.68
Ishii test	annual	13881.77	3470.76	9.6221	0.6210	1442.70	5588.97
MSRA-7	biennial	14030.25	3619.24	9.4975	0.4964	1477.25	7290.52
Calf circumference	annual	14160.75	3749.75	9.6313	0.6302	1470.29	5950.48
MSRA-7	annual	17147.98	6736.97	9.7257	0.7246	1763.17	9297.93

*Incr *incremental, *Eff * effectiveness, *CE* Cost-effectiveness, *ICER * incremental cost-effectiveness ratio, *QALY * quality-adjusted life-years

**Fig. 2:**
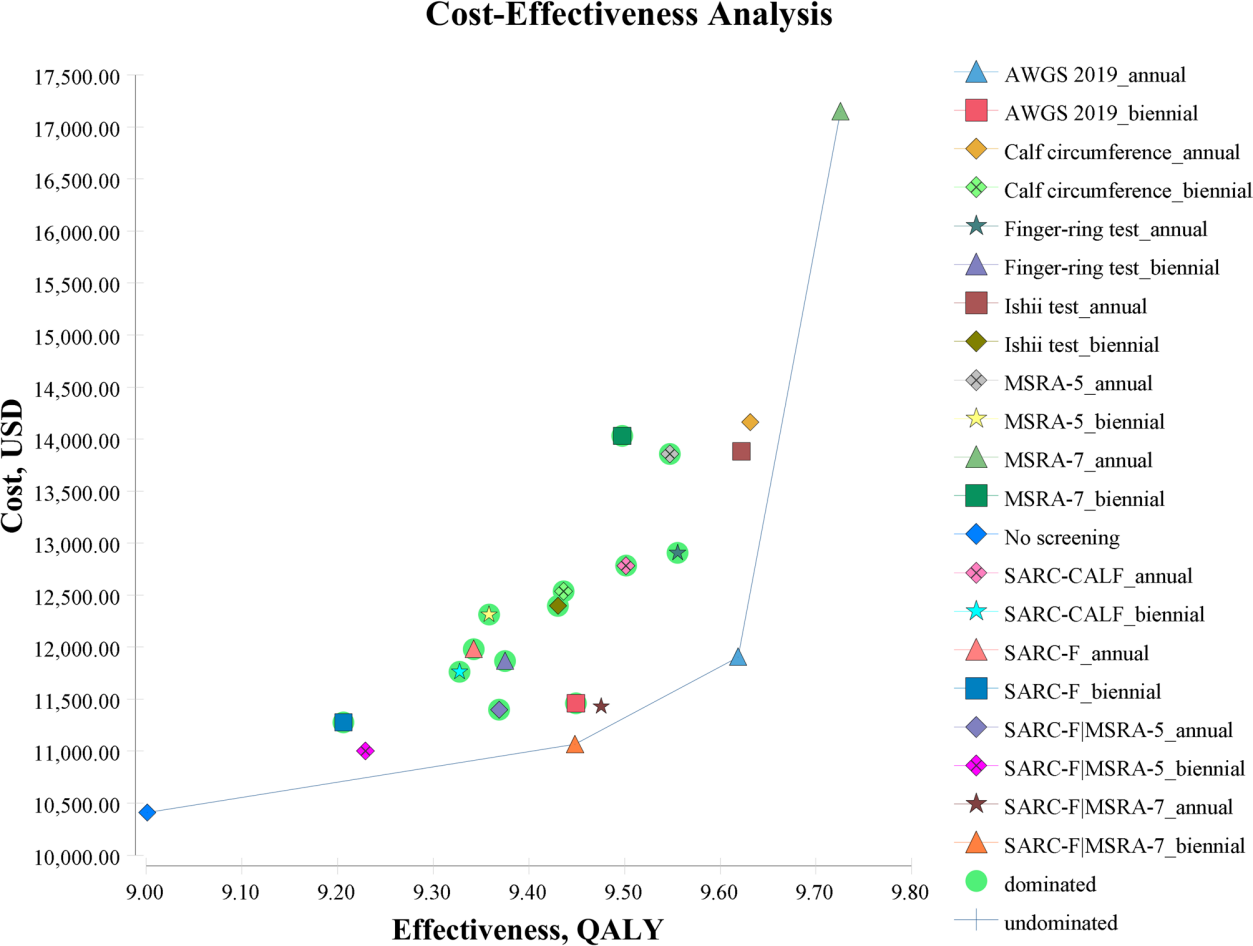
Cost-effectiveness plane of sarcopenia screening strategies

The scatter points in the bottom right corner of the graph were connected to form a cost-effectiveness frontier curve (Fig. 2), representing dominant strategies. Among them, the biennial SARC-F|MSRA-7, annual AWGS 2019, and annual MSRA-7 screening were dominant strategies. When using the per-capita GDP for China ($12,551.49) as the willingness-to-pay threshold, the preferred strategy was the annual MSRA-7 screening.

### Sensitivity analysis

#### Deterministic sensitivity analysis

Taking 3 times the per-capita GDP for China ($37,654.49) as the willingness-to-pay threshold, the discount rate ranged from 0.01 to 0.05. The ranges of variables such as the prevalence and recovery rate of sarcopenia, sensitivity and specificity of screening tools were based on the 95% confidence interval (CI). While a change of plus or minus 10% [45]for the original values were used for the remaining parameters.

**Fig. 3:**
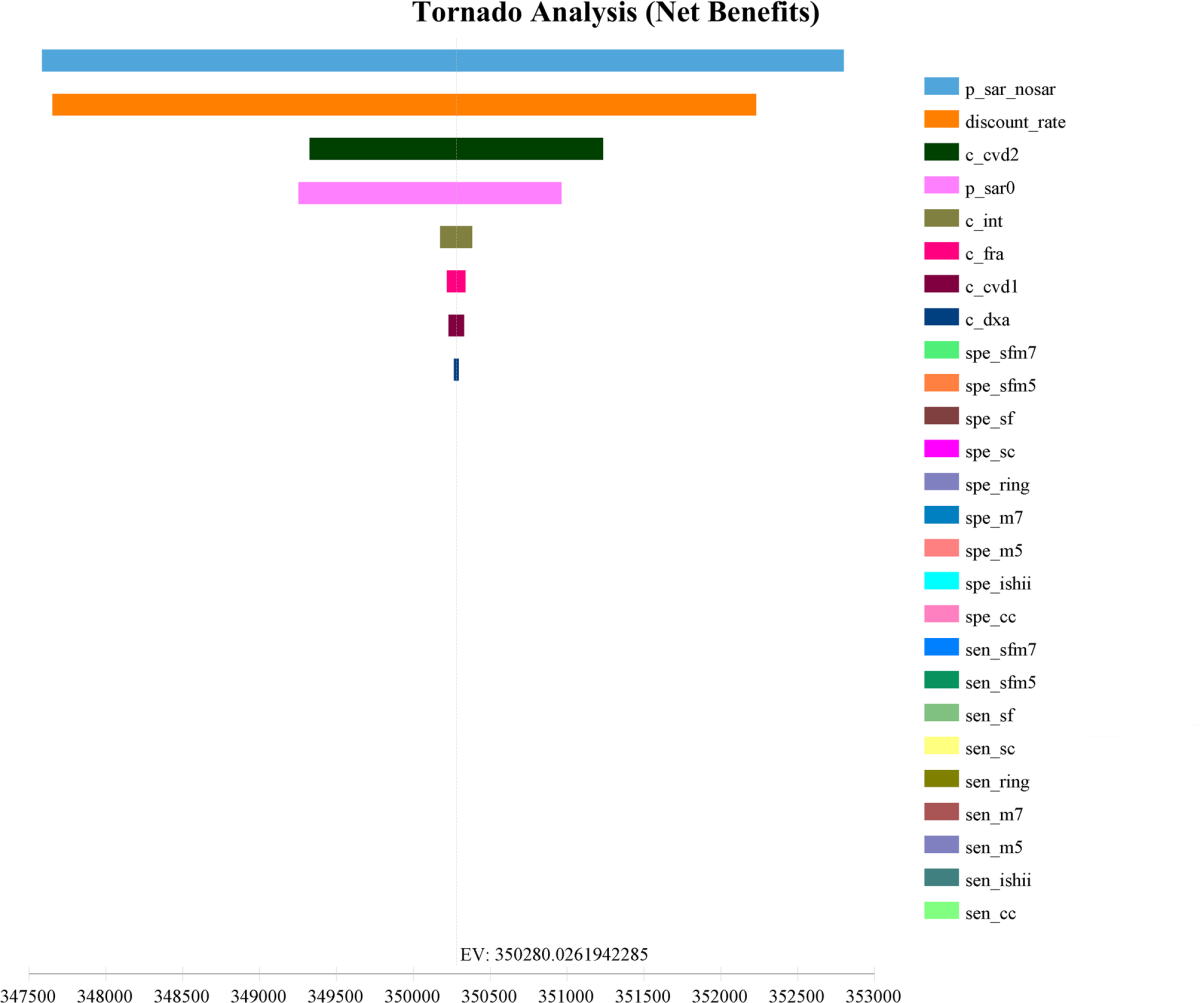
Deterministic sensitivity analysis using tornado diagram

The results showed that the recovery rate of sarcopenia (49.6%) and the discount rate (38.3%) had a significant influence on net benefits (Fig. 3). However, the ranking of ICER remained unchanged across five levels of the recovery rates of sarcopenia and the discount rates (Supplemental Fig. 1 and Fig. 2), which indicated the robustness of our main results.

#### Probabilistic sensitivity analysis

**Fig. 4:**
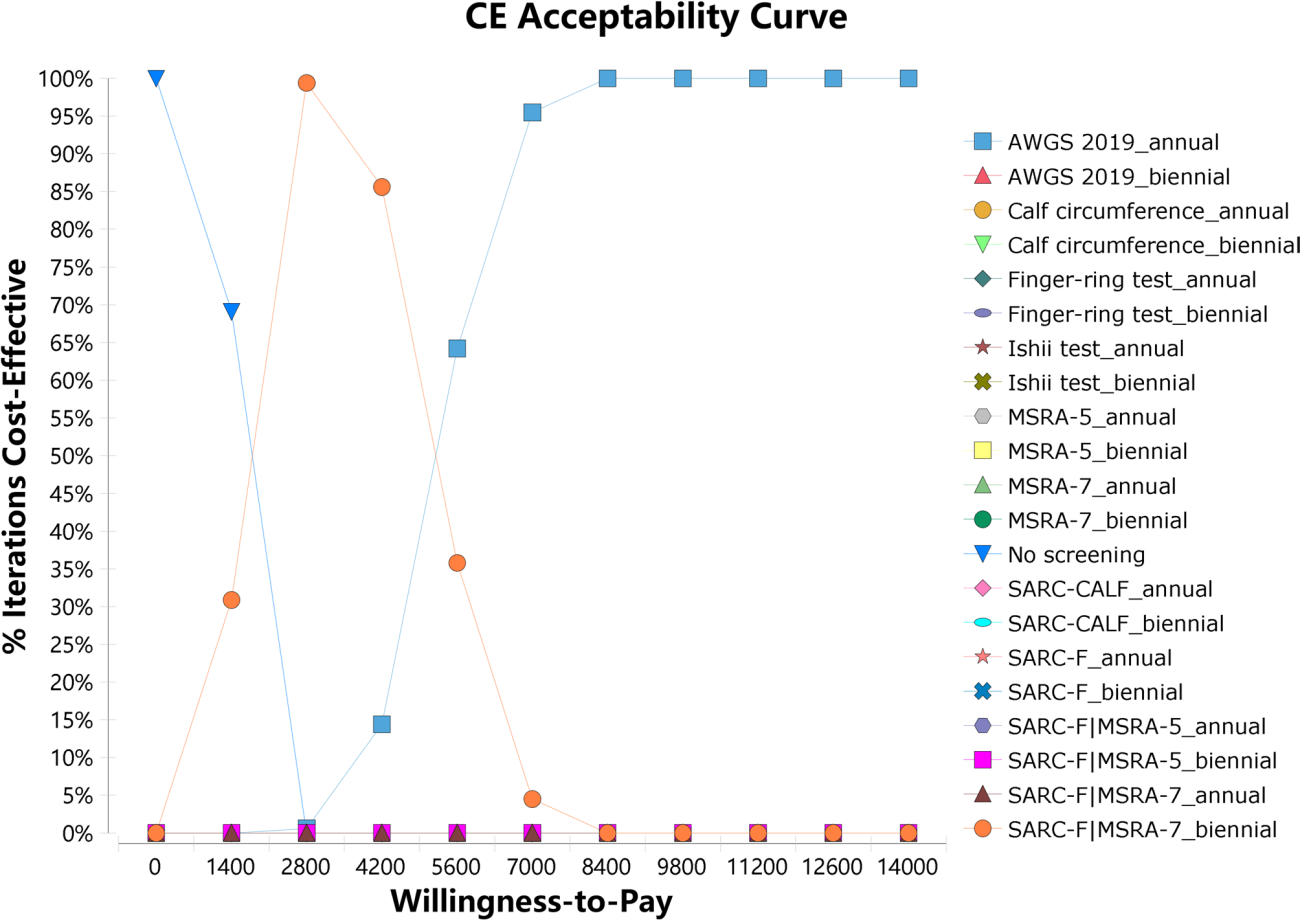
Cost effectiveness acceptability curve of sarcopenia screening strategies

The CEAC showed that the probability of cost-effectiveness increased for the annual AWGS 2019 screening strategy, while decreased for no screening with the willingness-to-pay threshold increasing. The probability of cost-effectiveness for the biennial SARC-F|MSRA-7 screening strategy initially increased and then decreased. When the threshold reached $2800, the probability of cost-effectiveness for the biennial SARC-F|MSRA-7 screening strategy increased to 98.9% and then decreased. The probability of cost-effectiveness for the annual AWGS 2019 screening strategy reached 100% and remained stable, when the threshold increased to $9800 (less than $12551.49), (Fig. 4).

## Discussion

Compared with no screening, all screening strategies for sarcopenia were highly cost-effective, with ICER less than the per-capita GDP for China ($12,551.49). The most cost-effective screening strategy was the biennial SARC-F|MSRA-7 screening, followed by the annual SARC-F|MSRA-7 screening, biennial AWGS 2019 screening, and annual AWGS 2019 screening. Among the four screening strategies, the annual AWGS 2019 screening gained the highest QALYs (9.62 years) and had a 100% probability of cost-effectiveness when the willingness-to-pay threshold reaches $9800. Therefore, we recommended the annual AWGS 2019 screening for sarcopenia screening among community-dwelling older persons in China. Additionally, the results of our study showed that the biennial SARC-F|MSRA-7 screening strategy had a higher probability of cost-effectiveness (98.9%) when the threshold reached $2800. Therefore, in budget-constrained settings or areas without DXA equipment, biennial SARC-F|MSRA-7 screening for community-dwelling older persons individuals was worth considering.

The AWGS 2019 was the gold standard for diagnosing sarcopenia in Asian populations. It improved upon AWGS 2014 by optimizing the thresholds for various assessments and provided specific diagnostic strategies for community-dwelling older persons. The complete strategy included case identification (screening positive for calf circumference, SARC-F, or SARC-CALF), assessment of muscle strength and physical performance, and measurement of appendicular skeletal muscle mass using Dual-energy X-ray absorptiometry (DXA) or bioelectrical impedance analysis (BIA). DXA was a commonly used imaging technique for assessing muscle mass due to its high accuracy [46]. The AWGS 2019 screening strategy consisted of two steps. The first step involved measuring grip strength and gait speed to determine muscle strength and physical performance. If the results of the first step were positive, DXA was performed to measure appendicular skeletal muscle mass [8]. Compared with other screening strategies, AWGS 2019 could accurately identify sarcopenia and facilitate timely interventions, leading to higher effectiveness than other strategies. However, DXA testing was relatively expensive and immobile, which would limit its widespread use in community settings. In such cases, BIA, an inexpensive and portable tool, could be considered as an alternative [46].

SARC-F and MSRA-7 both had significant disadvantages as standalone screening strategies for sarcopenia. SARC-F had the lowest sensitivity among all screening strategies, making it prone to underdiagnosing individuals at high risk. MSRA-7 had the lowest specificity among all screening strategies, leading to greater false positive cases and adding substantial additional costs for interventions. These two strategies had higher ICER compared with no screening, ranging from $4220.83 to $9297.93. Using SARC-F and MSRA-7 in combination could improve the sensitivity of sarcopenia screening, which might be more suitable for initial screening. In this study, this combined strategy had the lowest ICER (biennial: $1461.52; annual: $2147.82) and was considered as the most cost-effective option. Considering the large population size and the uneven economic development across regions in China, screening could still impose a significant economic burden even if the cost per additional QALY was relatively low. Therefore, the biennial SARC-F|MSRA-7 screening was an alternative cost-effective strategy.

The AWGS 2019 consensus on sarcopenia defined three sarcopenia statuses, namely possible sarcopenia, sarcopenia, and severe sarcopenia [8]. Since the proportion of severe sarcopenia among the older persons was relatively low (approximately 3%) [26], this study combined severe sarcopenia with sarcopenia into one group. There were limited researches on the transition probabilities between different health statuses of sarcopenia, with observation periods ranging from 1 to 10 years. Murphy et al. found dynamic transitions between different sarcopenia states [47], while a study in Switzerland suggested the possibility of bidirectional transitions in the early stages of sarcopenia [48]. In Chinese community-dwelling older persons, it was observed that within one year, the probability of transitioning from no sarcopenia to sarcopenia was 3.4%, the probability of sarcopenia recovery was 15.7%, and the probability of maintaining sarcopenia was 71.5% [26].

The observed differences in QALYs could be driven by several factors. Firstly, the sensitivity and specificity of screening tools may have an impact on respective QALYs of different screening strategies [49]. In our study, the SARC-F has a low sensitivity, it may lead to false negatives and delay the intervention opportunity. MSRA-5 has a low specificity, which may lead to false positives and increase unnecessary anxiety and medical expenses. Whether it is low sensitivity or low specificity, it will eventually affect the health of the older persons, reduce their quality of life, and lead to lower QALYs. Secondly, high-cost screening strategies may reduce their cost-effectiveness and affect the assessment results of QALYs [50], Our research showed that AWGS 2019 has high screening accuracy, but its screening cost is significantly higher than that of other strategies. Therefore, its QALY is not the highest. In addition, Screening intervals may be another factor to influence the benefits of screening strategies. The cost-effectiveness analysis showed that for each screening tool, the QALYs obtained with one year interval is higher than that of two years. The longer interval may lead to later detection of sarcopenia, thus reducing the intervention effect and life quality [51].

At present, there is only one study in Iran on the health economics evaluation of screening strategies for sarcopenia in the older persons. Similar to our findings, all screening strategies were cost-effectiveness, with annual screening based on EWGSOP 2010 being the most cost-effective [25]. Compared with previous study, our study considered more diverse range of screening tools (e.g. MSRA-5, Calf circumference, Ishii test, Finger-ring test, SARC-CALF, AWGS 2019), and intervals (one year and two years), and constructed 20 screening strategies. Except for states including three states, sarcopenia, sarcopenia with CVD, and death [25], the natural history model of sarcopenia constructed in this study included two additional states, no sarcopenia and developed CVD, which allowed for a more accurate simulation of the progression of sarcopenia. These advantages greatly expand the scope of exploration for comparative discovery of the best screening strategy, providing a more comprehensive health economic evaluation for sarcopenia screening.

This study has some limitations. Firstly, in this study, only CVD was incorporated into the natural history model of sarcopenia. Due to the simplification of the model, this study has not yet included other diseases related to sarcopenia, which may lead to an underestimation of the screening effect. Sarcopenia and CVD often coexist and influence each other, creating a vicious cycle [52]. There are multiple connections between CVD and sarcopenia, including common pathophysiological mechanisms and mutually influencing disease development processes. Inflammatory factors can promote the breakdown of muscle protein, inhibit muscle protein synthesis, and at the same time damage vascular endothelium, accelerating the process of atherosclerosis [53]. A decrease in muscle mass and strength can lead to a decline in physical activity, reduced energy expenditure, and an increased risk of obesity, insulin resistance, and metabolic syndrome, all of which elevate the risk of CVD [54]. Additionally, CVD was the leading cause of death among older persons [55]. The coexistence of sarcopenia and CVD will have a more severe combined impact on the health status of the older persons. Other sarcopenia-related outcomes such as cognitive impairment, diabetes, or hearing loss [1] would be considered in the future study to conduct a more comprehensive model and health economics analysis. Secondly, current research on health economics evaluation of sarcopenia screening was still in the preliminary stage, and certain age-related parameters were unavailable, such as age-specific prevalence and recovery rates of sarcopenia, age-specific utility values for different health states, etc. However, the parameters used in this study had extensively utilized local data, making the research model more suitable for Chinese older persons. Future studies are needed to collect more age-specific data from community samples, refining model and make projections more precise. Thirdly, considering that the sensitivity and specificity parameters for the SARC-F|MSRA-7 and SARC-F|MSRA-5 strategies were derived from older persons within a hospital setting [36], the main results might be biased. Nevertheless, one-way DSA showed that the ranking of ICER remained stable for each screening strategy within their respective ranges of variation, which indicated the robustness of our findings. In the future study, appropriate data should be collected in the community-dwelling older persons in order to conduct a more accurate health economics analysis. Additionally, the economic evaluation focused solely on China. The generalization of modelling results is limited, and the results cannot be applied to other countries without cautions due to differences in healthcare costs and demographics.

Furthermore, this study assumed 100% for screening participation rate, intervention rate, and fracture healing rate. These assumptions might deviate from actual circumstances and potentially overestimate the effectiveness of the screening. On the one hand, each screening strategy requires a fixed investment of costs, such as equipment, consumables, and human resources. A low participation rate directly leads to an increase in the unit screening cost and reduces the economic effect [56]. On the other hand, a low participation rate means that a large number of the target population have not been screened, and diseases may not be discovered until the advanced stage, significantly increasing the difficulty of treatment, while the health benefits of early intervention are greatly reduced [57]. In addition, the screening resources that have been invested will not be fully utilized due to the low participation rate, resulting in a waste of resources. Low intervention compliance will reduce the benefits of screening, which hindered rapid recovery of sarcopenia patients, increased the risks of adverse outcomes, and the difficulty and cost of subsequent treatment [58]. Future researches are needed to investigate realistic rates of screening participation and intervention compliance, thus analyzing the health economics effects of each strategy under different scenarios.

## Conclusion

This study conducted a health economics evaluation of multiple strategies for sarcopenia screening among community-dwelling older persons. The results showed that all screening strategies were highly cost-effective, with the biennial SARC-F|MSRA-7 strategy being the most cost-effective. Combining the PSA results, we recommended the annual AWGS 2019 screening among community-dwelling older persons in China.

## Supplementary Information

Supplementary Material 1.

## Data Availability

All data generated or analysed during this study are included in this published article [and its supplementary information files]. The datasets used and/or analysed during the current study are available from the corresponding author on reasonable request.
