# Supplementary material for: Health economics evaluation of screening for sarcopenia among community-dwelling older persons
Source: BMC Public Health. 2025 Nov 7;25:3827. doi: 10.1186/s12889-025-25263-x (PMC12595691; doi:10.1186/s12889-025-25263-x)
Supplement: Supplementary file 1 — Supplementary Material 1. [file 12889_2025_25263_MOESM1_ESM.docx]

Supplemental Materials

Supplemental Table 1. The incidence, prevalence, and mortality rates of CVD and fall.

Supplemental Figure 1 Incremental cost-effectiveness ratio based on sarcopenia recovery rate.

Supplemental Figure 2 Incremental cost-effectiveness ratio based on discount rate.

Supplemental Table 1 The incidence, prevalence, and mortality rates of CVD and fall

| Age group | CVD | | |  | Fall | | |
| --- | --- | --- | --- | --- | --- | --- | --- |
|  | Incidence  rate | prevalence | Mortality  rate |  | Incidence  rate | prevalence | Mortality  rate |
| 60-64 | 1886.41 | 19157.72 | 362.21 |  | 2477.78 | 9954.02 | 9.30 |
| 65-69 | 2569.94 | 26695.25 | 644.07 |  | 2741.77 | 12235.47 | 12.53 |
| 70-74 | 3382.87 | 34481.05 | 1295.77 |  | 3298.61 | 15538.37 | 22.48 |
| 75-79 | 4362.78 | 42247.97 | 2367.04 |  | 4876.72 | 20192.65 | 46.12 |
| 80-84 | 5989.45 | 49501.74 | 4692.24 |  | 7750.26 | 28390.95 | 112.35 |

*Note*. CVD=cardiovascular diseases; Sourced from GBD 2019^[1]^, the unit is per 100,000 population.


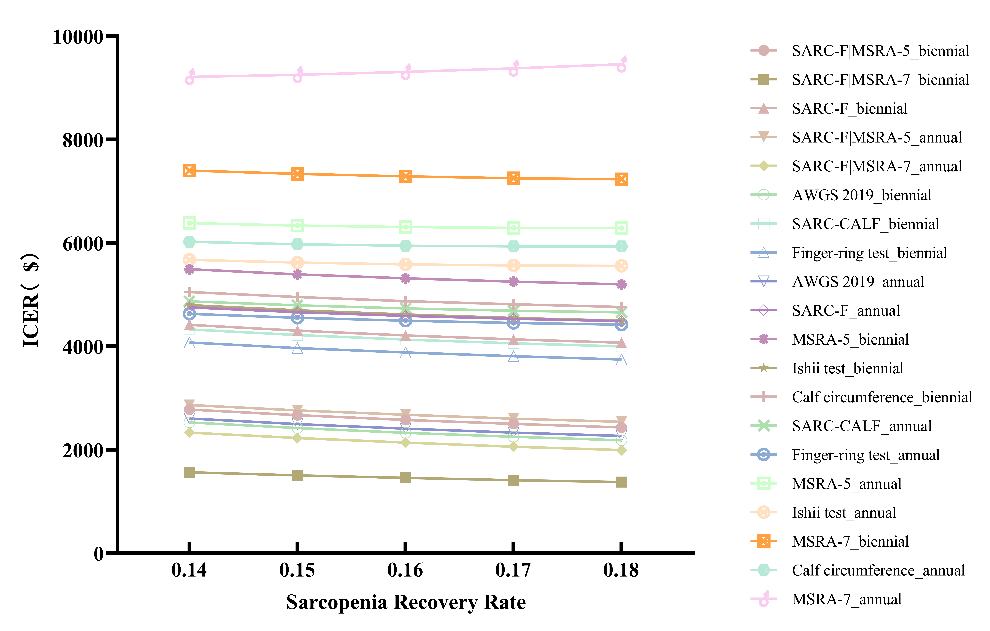


Supplemental Figure 1 Incremental Cost-Effectiveness Ratio Based on Sarcopenia Recovery Rate


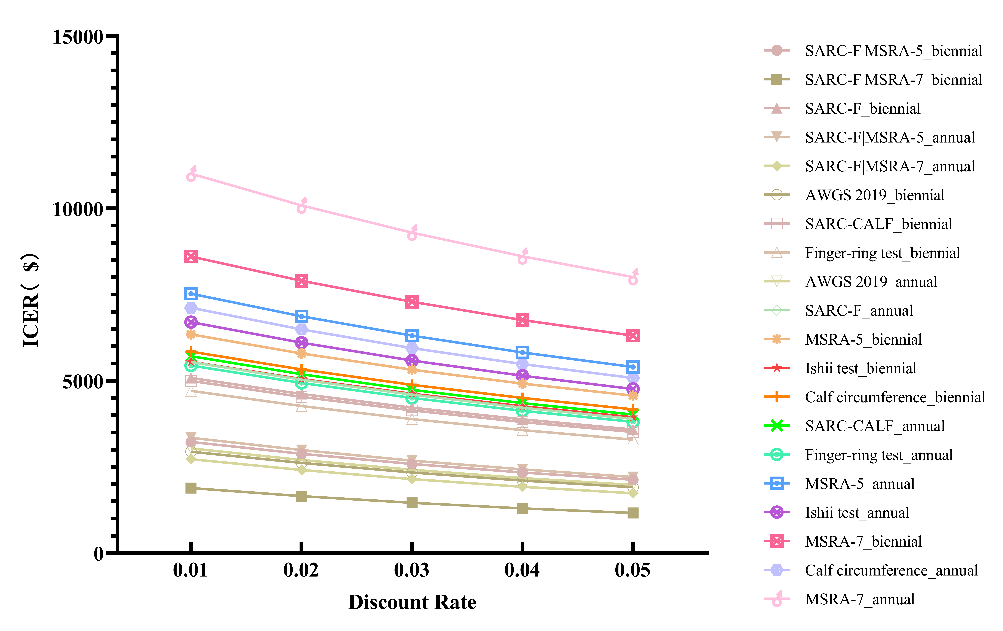


Supplemental Figure 2 Incremental Cost-Effectiveness Ratio Based on Discount Rate

**References**

1. Global Burden of Disease Collaborative Network. Global Burden of Disease Study 2019 (GBD 2019) results. 2024 Accessed: 4/30/2024. Available from: http://ghdx.healthdata.org/gbd-results-tool.
